# Three-Year Outcomes of Patients with Neovascular Age-Related Macular Degeneration Treated with Aflibercept under the National Health Insurance Program in Taiwan

**DOI:** 10.1155/2020/4538135

**Published:** 2020-02-21

**Authors:** Kang-Jung Lo, Jin-Yu Chang, Hsin-Yi Chang, Shih-Hwa Chiou, De-Kuang Hwang, Shih-Jen Chen

**Affiliations:** ^1^Department of Ophthalmology, Taipei Veterans General Hospital, Taipei, Taiwan; ^2^Department of Ophthalmology, Shin Kong Wu Ho Su Memorial Hospital, Taipei, Taiwan; ^3^Institute of Clinical Medicine, National Yang-Ming University, Taipei, Taiwan; ^4^Institute of Pharmacology, National Yang-Ming University, Taipei, Taiwan; ^5^Department of Ophthalmology, School of Medicine, National Yang-Ming University, Taipei, Taiwan

## Abstract

**Purpose:**

To observe and analyze the long-term outcomes of patients with neovascular age-related macular degeneration (nAMD) treated with aflibercept monotherapy under the National Health Insurance (NHI) program in Taiwan.

**Methods:**

This retrospective observational study was conducted at Taipei Veterans General Hospital. Patients with naive nAMD who were treated with aflibercept and followed for more than 3 years were reviewed. The better eye was enrolled if both eyes were affected. Visual acuity (VA) and central macular thickness (CMT) were recorded for 3 years. The lost-to-follow-up rate, number of injections, and predictive factors for visual outcomes were analyzed.

**Results:**

Ninety-nine eyes in 99 patients were followed up for 3 years. The mean age at onset of nAMD was 82.8 ± 9.26 years, and 65% of the patients were male. Compared with initial visual acuity, 5 (5.1%) of our patients improved their vision for 3 or more lines after 3 years of follow-up, 11 (11.1%) of our patients improved for 1 to 3 lines, 62 (62.6%) patients remained their vision with 1 line or less changes, 15 (15.2%) patients lost their vision for 1 to 3 lines, and 6 (6%) patients lost their vision for 3 or more lines. The CMT was 359 ± 180 *µ*m before treatment and 259 ± 98 after 3 years (*p* < 0.001). The mean number of injections was 4.63 ± 1.91 in the first year, 2.13 ± 2.2 in the second year, and 1.42 ± 1.79 in the third year. Multivariate analysis showed that final VA was significantly associated with VA at year 1, the presence of retinal pigment epithelial detachment at year 1, and receiving more than four injections in the first year. Final CMT was only significantly associated with CMT at year 1.

**Conclusion:**

After 3 years of treatment under the NHI program in Taiwan, 21.2% of the patients with nAMD still had a visual decline despite good anatomical outcomes. More aggressive treatment or other strategies should be used for patients who may have a poor prognosis.

## 1. Introduction

Neovascular age-related macular degeneration (nAMD) was a leading cause of visual impairment without optimal treatment in developed countries for decades [1]. However, the introduction of intravitreal injections of antivascular endothelial growth factor (anti-VEGF) agents has shown promising results in recent years. Patients in previous clinical trials have been treated with ranibizumab or aflibercept based on a fixed monthly or bimonthly dosing protocol in the first 2 years, which is impractical in a real-world setting [2–5]. To balance the burden of frequent clinic visits for injections and costs/benefits of the treatment, regimens including pro re nata (PRN), treat-and-extend (T&E), and observe-and-plan have been proposed in recent years or used in real-world clinical practice [6–11].

However, nAMD treatment is a continuous process. In long-term results, a decline in VA to worse than baseline has been reported after a few years during the extension phase of previous trials, such as the MARINA study and CATT trials [12, 13] and a database observational study (Fight Retinal Blindness! Registry (FRB) and AURA study) [14, 15] in patients treated with ranibizumab. In the FRB study, a mean decline of 2.6 letters in the Early Treatment Diabetic Retinopathy Study (ETDRS) at the end of 7 years was noted, which treated patients with six injections in the first year followed by five injections annually in consecutive years [12]. In the AURA study, VA improved by +2.4 and +0.6 EDTRS letters with a mean of 5.0 and 2.2 injections in the first and second years, respectively [15]. The visual outcomes of real-world data have been noninferior to these trials if the patients received more injections during the observation periods [16].

The government in Taiwan launched the National Health Insurance (NHI) program in 1995, and currently it covers more than 99% of residents and health care utilities in Taiwan. The Bureau of NHI approved ranibizumab and aflibercept to treat nAMD in 2011 and 2014, respectively. Copayments are not required; however, a limited number of doses are reimbursed and switching agents are not permitted [17]. The aim of this study was to investigate the results of long-term outcomes of patients with nAMD treated with aflibercept under the NHI program in Taiwan.

## 2. Materials and Methods

### 2.1. Study Design, Patient Selection, and Treatment Intervention

This retrospective study was approved by the Institutional Review Board of Taipei Veterans General Hospital in Taiwan, and all research studies followed the tenets of the Declaration of Helsinki. We reviewed medical records of all patients who visited Taipei Veterans General Hospital from 2014 to 2019 with a diagnosis of treatment-naïve nAMD and who were eligible to receive intravitreal injections of aflibercept under the NHI program. The inclusion and exclusion criteria were as follows [17]:

1. Age ≥50 years and diagnosed with nAMD based on fundus photography, fluorescence angiography, and optical coherence tomography.
2. Best-corrected VA between 20/40 and 20/400, as tested by Snellen equivalent.
3. Patients with choroidal neovascularization due to etiologies other than nAMD (such as high myopia or uveitis) or advanced macular scarring, subretinal fibrosis, and geographic atrophy were excluded.
4. Three doses of anti-VEGF agents were allowed for the first application, with an additional four doses permitted if the disease activity responded to the treatments. For each eye, a lifetime maximum of seven doses could be reimbursed.
5. Changing or switching between the two anti-VEGF agents was not permitted.
6. All patients had to pay for the anti-VEGF medication if their application was not approved or if they had already received seven reimbursed injections.

If both eyes of the same patient were successfully covered by the NHI program, we only enrolled the eye which was diagnosed first. All enrolled patients were followed up for 3 years after the first aflibercept injection. Patients who withdrew and were followed up for less than 3 years were recorded, but excluded from the final analysis.

### 2.2. Treatment Protocol

Most of the patients in this study received 3 consecutive monthly injections during the initial loading phase. The treatment regimens were decided by doctors based on patients' clinical presentations. Most of them received injections under a PRN regimen in which they received treatment when their visual acuity dropped for more than 2 lines in a Snellen chart compared with the previous visit without developing other ocular diseases, or presenting any intraretinal or subretinal fluid in optical coherence tomography (OCT) exam. All patients paid for full amount of medications and treatments after they depleting the injections reimbursed by NHI. The follow-up frequency of each patient was decided by a doctor individually based on their clinical presentation and response to the treatment.

### 2.3. Outcome Measurements

The primary outcome in this study was the final VA 3 years after the initial injection. The best corrected VA was converted to logMAR (logarithm of the minimum angle of resolution). We also analyzed the difference in VA before and after treatment. Central macula thickness (CMT) was also measured after the initial injection. To record CMT, an OCT scanner, Avanti RTVue XR (OptoVue, Fremont, CA, USA), was used for this study. Fluorescence angiographies were checked for all patients to confirm the diagnosis and activity of nAMD before initiation of therapy. Repeated fluorescence angiography was checked after 3 anti-VEGF injections if the doctor thought it was necessary. Indocyanine green angiographies were done for those patients with suspicious signs of polypoidal choroidal vasculopathy (e.g., double-layer sign, high elevated RPED, massive subretinal or subretinal pigment, epithelial hemorrhage, etc.).

Visual outcomes were categorized into 5 groups: vision improved for 3 or more lines in Snellen chart after 3 years of follow-up, improved for 1 to 3 lines, stable as vision changes between final and initial tests were within 1 line, lost their vision for 1 to 3 lines, and lost their vision for 3 or more lines.

### 2.4. Associated Factor Analysis

The presence of subretinal fluid (SRF), intraretinal cyst (IRC), and retinal pigment epithelial detachment (RPED) at baseline and each follow-up visit was documented in the medical records and reconfirmed independently by two investigators (K.J. Lo and D.K. Hwang).

### 2.5. Statistical Analysis

Statistical analysis was performed using SPSS software version 22.0 (SPSS, Inc., Chicago, Illinois, USA). A *p* value less than 0.05 was considered to be statistically significant in all analyses. VA and CMT measurements between baseline and follow-up visits were analyzed using the paired Student's *t*-test. To evaluate the potential predictive factors for visual outcomes and CMT at the third year, independent variables including age, sex, VA, and CMT at baseline, month 3, year 1, and year 2; SRF, IRC, and RPED at baseline and year 1; total number of injections at year 1 and year 3 were analyzed in a stepwise multiple linear regression model. Youden's index was used to calculate the ideal total number of injections at year 1, 2, and 3 in the patients who improved by 3 or more lines in Snellen chart at year 3.

## 3. Results

### 3.1. Patients' Characteristics

A total of 180 eyes of 180 patients were identified initially. Among them, 37 (21%), 30 (17%), and 14 (7%) patients were lost to follow-up before the first, second, and third years, respectively. Of the 99 patients who completed all 3 years of follow-up, 65 were male and 34 were female, with a mean age of 82.77 ± 9.26 years. The total number of clinics in 3-year follow-up ranged from 20 to 34 times, with a median of 25 times. Among these patients, 27 patients were subclassified as classic CNV (choroidal neovascularization), with 46, 14, and 10 patients were subclassified as occult CNV, RAP (retinal angiomatous proliferation), and PCV (polypoidal choroidal vasculopathy), respectively. The remaining 2 patients could not be subclassified clearly due to the poor quality of fluorescence angiography before enrolling.

### 3.2. Visual Outcomes

**Figure 1:**
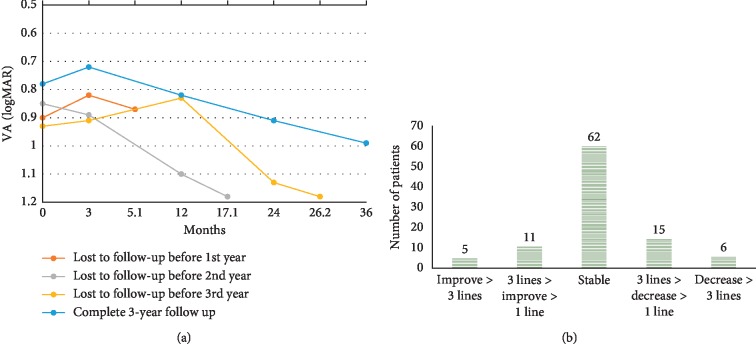
(a) Visual outcome at month 3, year 1, year 2, and year 3 in lost-to-follow-up before year 1, year 2, and year 3 and complete 3-year follow-up groups. (b) The third-year visual outcome compared to baseline in the complete 3-year follow-up group.

The visual outcomes of the patients who completed 3 years of follow-up and in those lost to follow-up are shown in Figure 1(a). The average follow-up periods were 5.1 ± 2.8, 17.1 ± 3.4, and 26.2 ± 2.1 months in those lost to follow-up before the first, second, and third years, respectively. All of the patients who were lost to follow-up had stabilized vision initially and then gradually decreased until they dropped out. A better baseline VA and relatively flatter decline slope were noted in those who completed 3 years of follow-up. The average VA in logMAR was 0.78 ± 0.44 at baseline and 0.99 ± 0.61 after 3 years of follow-up. Comparing with the initial visual acuity, 5 (5.1%) of our patients improved their vision for 3 or more lines after 3 years of follow-up, 11 (11.1%) of our patients improved for 1 to 3 lines, 62 (62.6%) patients remained their vision with 1 line or less changes, 15 (15.2%) patients lost their vision for 1 to 3 lines, and 6 (6.1%) patients lost their vision for 3 or more lines (Figure 1(b)). In addition, 4% of patients improved for more than 0.3 in logMAR.

No patients experienced ocular (e.g., endophthalmitis, retinal detachment, and enlargement of significantly geographic atrophy) and systemic (e.g., cerebrovascular accidents or cardiovascular diseases) side effect during the follow-up period.

### 3.3. CMT Outcome

**Figure 2:**
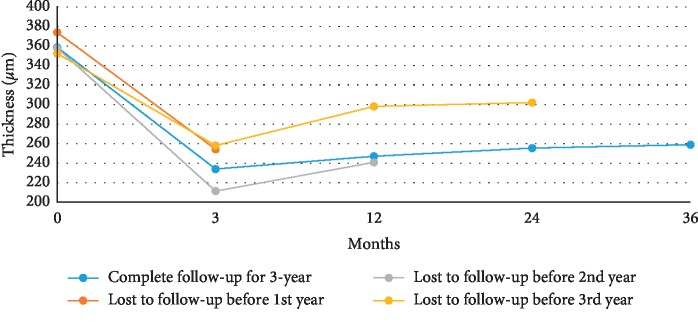
Central macula thickness at month 3, year 1, year 2, and year 3 in lost to follow-up before year 1, year 2, and year 3 and complete 3-year follow-up groups.

The CMT values in those who completed 3 years of follow-up and those lost to follow-up are shown in Figure 2. A significant decrease in CMT in the treated eyes was noted, with an average thickness of 359 ± 180 *μ*m at baseline and 234 ± 59 *μ*m at the third year (*p* < 0.001).

### 3.4. Number of Injections

**Figure 3:**
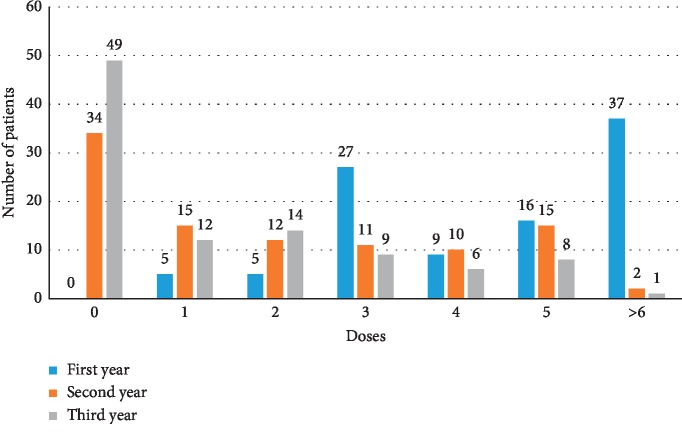
Number of injections at years 1, 2, and 3 in the complete 3-year follow-up group.

The number of injections and percentage of patients who received a different number of injections in each year in those who completed 3 years of follow-up are shown in Figure 3. The average numbers of injections were 4.63 ± 1.91, 2.13 ± 2.12, and 1.42 ± 1.79 in the first, second, and third years, respectively. An average total of 8.16 ± 4.57 doses were given over 3 years. Overall, 37% of the patients received over six doses, and 27% of the patients received three doses in the first year. In the second year, only 10% to 15% received one to five doses and 3% (3/99) received over six doses. The distribution of the number of injections in the third year was totally different compared with the previous two years, and almost half (49%) of the patients did not receive any further injections, and 75% received fewer than two doses.

### 3.5. Predictive Factors for Visual Outcome

The predictive factors which affected the third-year visual outcomes in those who completed 3 years of follow-up are shown in Table 1. In univariate analysis, younger age (*p*=0.003), better VA at baseline (*p* < 0.001), at month 3 (*p* < 0.001), at year 1 (*p* < 0.001), and at year 2 (*p* < 0.001) and absence of IRC at baseline (*p*=0.004), a greater number of injections in the first year (*p*=0.029), and within three years (*p* < 0.003) were associated with a better visual outcome at the third year. However, after stepwise multivariate analysis, only better VA at year 1 (*p* < 0.001), absence of RPED at year 1 (*p*=0.011), and receiving more than four injections (*p* < 0.001) at year 1 were significantly associated with a better visual prognosis at the third year.

**Figure 4:**
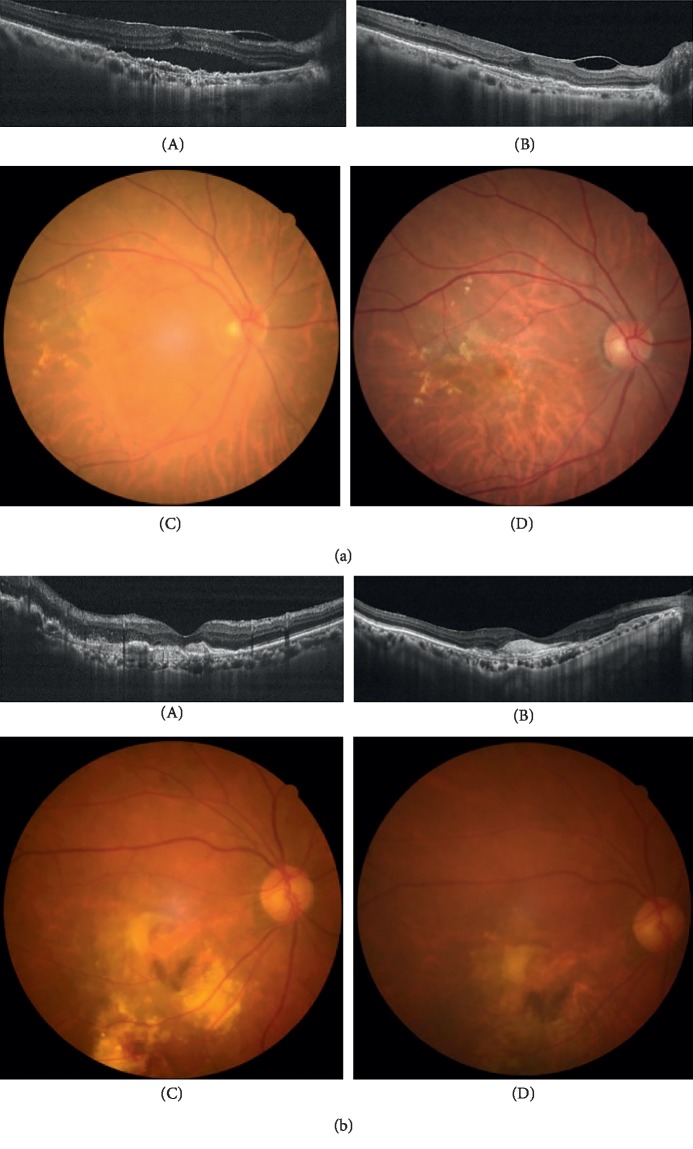
(a) An 80-year-old female who was diagnosed nAMD during her first visit. After receiving 7 doses (6 doses in the first year) of aflibercept injections within three years, her vision improved from 0.52 to 0.10 in logMAR. Fundus photography and OCT before treatment (A, C) and three years after treatment (B, D). (b) An 87-year-old female who was diagnosed nAMD during her first visit. After receiving 3 doses (3 doses in the first year) of aflibercept injections within three years, her vision decreased from 1 in logMAR to hand motion. Fundus photography and OCT before treatment (A, B) and three years after treatment (C, D).

To identify the factors associated with a better visual outcome at 3 years, the patients who had an improvement in VA by more than two lines in the Snellen chart compared to baseline VA were analyzed. We analyzed the number of injections in each year of this group and used Youden's index to calculate the cutoff value for a better visual outcome. We found that the patients who received more than 4.5 (*p* < 0.05), 3.5 (*p*=0.494), and 4.5 (*p* < 0.001) doses in the first, second, and third years, respectively, achieved better visual outcomes (Figure 4 shows clinical pictures of patients before and after 3 years of follow-up).

### 3.6. Predictive Factors for CMT

The predictive factors for CMT at 3 years in those who completed 3 years of follow-up are shown in Table 2. A thinner CMT at month 3, year 1 and year 2, and the absence of IRC at year 1 were associated with a thinner CMT at year 3 after univariate analysis. In stepwise multivariate analysis, a thinner CMT at year 1 was the only predictive factor for a thinner CMT at the third year.

## 4. Discussion

This study demonstrated the real-world situation of treating nAMD with anti-VEGF monotherapy under the NHI program in Taiwan. In this study, 99 patients completed 3 years of follow-up. Sixteen patients had improved VA by over one line in the Snellen chart, while the vision in 62 patients remained stable and 21 patients experienced a decline in VA by over one line in the Snellen chart at the end of the third year. The mean VA improved from baseline (0.78 ± 0.44 in logMAR) after three loading doses (0.72 ± 0.49 in logMAR), followed by a deterioration in visual outcome from year 1 to year 3 (0.82 ± 0.53, 0.91 ± 0.57, and 0.99 ± 0.61 in logMAR at the end of years 1, 2, and 3, respectively). The mean CMT decreased from baseline (359 ± 180 *μ*m) and maintained a stable thickness (234 ± 59 *μ*m, 247 ± 87 *μ*m, 255 ± 88 *μ*m, and 259 ± 98 *μ*m at the end of month 3, year 1, year 2, and year 3, respectively) throughout the 3 years. Although 79% of the patients had stable or improved vision, the average VA was still worse at the end of the third year.

Eleftheriadou et al. reported 3-year outcomes of treating nAMD with aflibercept [18]. In their study, VA improved from 54.4 ± 16.6 ETDRS letters to 61 ± 16.6 ETDRS letters and VA improved by 15 letters or more in 30.5% of the patients at the end of the third year. They administered 7.2 ± 1.8 injections in the first year, with a total of 15.9 ± 6.1 doses at the end of the third year. Traine et al. followed up nAMD patients treated with aflibercept for 4 years [19]. In their study, VA improved from 59.8 ± 16.9 letters to 64.2 ± 19.4 letters at the third year and 63.4 ± 20.4 letters at the fourth year, and they administered 7.7 ± 1.2 injections in the first year, followed by on average 4.4 ± 1.9 injections during the second to fourth years. Nishikawa et al. reported the 4-year outcomes of nAMD patients treated with aflibercept, and their results showed visual gains (logMAR) of 0.14, 0.13, 0.07, and 0.06 in years 1, 2, 3, and 4, respectively [20]. On average, these patients received 7.0 and 2.5 injections in the first and second years, respectively, followed by 2.7 injections each in the third and fourth years. The worse VA at the end of the third year in our study may mainly be attributed to an insufficient number of injections during the 3-year period. An average total of 8.16 ± 4.57 doses was given within 3 years, with an average of 4.63 ± 1.91, 2.13 ± 2.12, and 1.42 ± 1.79 injections in the first, second, and third years, respectively. Most of our patients paid for extra anti-VEGF doses after they had used all seven reimbursed aflibercept injections under the NHI program. The number of treatments was relatively few and visual outcomes were relatively poor in our patients. We hypothesized that there were two main factors influenced by the health insurance policy in Taiwan. Firstly, the reimbursement of anti-VEGF therapy required an approval of administrative application before the first injection, which usually took 2 to 3 weeks. This delaying of treatment may result in relatively poor visual outcome, thus, decreased patients' compliance to the therapy. Secondly, although many studies have shown that the aggressive therapeutic protocol such as fixed-dose or T&E regimen results in a better outcome than PRN regimen, ophthalmologists and patients tended to adopt the as-needed schedule with a limitation of reimbursing dosed (seven per eye). Besides, patients would not get the reimbursement if their vision was better than 20/40, worse than 20/400, or had a large macular scar.

Besides the number of injections, older age and worse baseline VA also influenced the final visual outcome. The mean age of our patients (around 83 years) was older than in other real-world nAMD studies (<80 years) [18–22]. Although age played a minor role in the final VA, other studies have reported that older age may be related to a poor visual outcome [23, 24]. The baseline VA (0.78 logMAR) in our study was inferior than in other real-world studies (around 60 EDTRS letters, equal to 0.5 logMAR) [18–22]. Adrian et al. reported out that a better initial VA may result in a better visual outcome [25]. This may partially explain why our visual prognosis was not as good as in other studies [18–22].

To further investigate the predictive factors that affected the visual outcome and CMT at the end of year 3, we analyzed age, sex, visual acuity, CMT, OCT characteristics of nAMD (presence of SRF, IRC, and RPED), and number of injections received in each year using a stepwise multiple linear regression model. Surprisingly, the final visual outcome was related to VA at the first year instead of baseline. This may explain why the patients who received over four injections in the first year had a better visual outcome in our study, which is consistent with other studies [18–20].

Kim et al. published a meta-analysis which included studies of patients treated with ranibizumab for nAMD [26]. Their results showed a mean change in VA of +1.1 in ETDRS letters at the end of the third year, with a mean of 6.3, 4.4, and 3.3 doses in the first, second, and third years, respectively. We also analyzed the number of injections in each year in the patients who had a visual improvement by more than three lines in the Snellen chart and used Youden's index to identify the number of doses associated with an improved final vision. We found that the patients who received over 4.5, 3.5, 4.5, and 12.5 injections in years 1, 2, and 3 and within 3 years, respectively, had a better visual prognosis. Previous studies have reported that the presence of RPED and IRC at baseline were related to worse VA outcomes [27, 28]. Lai et al. also reported that the presence of RPED and IRC at month 12 was related to final visual outcome in their 1-year follow-up study of anti-VEGF therapy in nAMD patients [29]. In our study, we found that the presence of RPED at year 1 may have been related to a worse visual outcome at the third year. In contrast to visual outcome, the third year CMT was only related to CMT at year 1.

The drop-out rates were 21%, 17%, and 7% before the first, second year, and third years, respectively. Mehta et al. analyzed the lost to follow-up rate in observational studies of anti-VEGF therapy in nAMD patients and reported a rate ranging from 17% to 34% at year 1 to 54% at year 5 [14]. Long-term outcomes of the FRB study showed a <10% drop-out rate in the first 2 years, increasing to 46% at the end of year 5 [16]. The lost-to-follow-up rate was 45% at the end of year 3 in our study; comparing with real-world studies in other countries, we have a relatively higher lost-to-follow-up rate. We found that there was a relatively higher lost-to-follow-up rate in patients with worse initial visual acuity, more systemic comorbidities, and poor treatment response to anti-VEGF therapy. This is not only a limitation of our study but also a barrier for achieving patients' optimal visual outcome in the real-world setting in Taiwan.

Furthermore, there are some limitations to this study. First, we only enrolled patients treated with aflibercept; therefore, we may have missed patients treated with ranibizumab. Second, the small sample size is another limitation as we only enrolled patients from a single referral hospital. Third, the high lost-to-follow-up rate may have led to bias in better visual prognosis since these patients may have had a poor visual prognosis and were not taken into consideration in the final outcome.

## 5. Conclusions

After 3 years of treatment under the NHI program in Taiwan, 21.2% of the patients with nAMD still had a visual decline despite good anatomical outcomes. To achieve better visual outcomes, more intensive treatment and more injections would definitely have been needed over the 3 years. Better best-corrected VA at year 1, absence of RPED at year 1, and receiving more than four injections at year 1 were good prognostic indicators for a better visual outcome at the third year.

## Figures and Tables

**Table 1:** Univariate and multivariate analysis for visual outcome at the third year in the complete 3-year follow-up group (*n* = 99).

*n* = 99	Univariate analysis		Multivariate analysis	
	*β*	*p* value	*β*	*p* value
Age	0.292	0.003^*∗*^	−0.046	0.469
Sex	0.133	0.189	0.104	0.085
Visual acuity				
Baseline	0.62	<0.001^*∗*^		
** **3^rd^ month	0.686	<0.001^*∗*^		
** **1^st^ year	0.765	<0.001^*∗*^	0.717	<0.001^*∗*^
** **2^nd^ year	0.89	<0.001^*∗*^		
Central macular thickness (CMT)				
** **Baseline	0.094	0.395		
** **3^rd^ month	−0.148	0.204		
** **1^st^ year	−0.027	0.807	−0.04	0.626
** **2^nd^ year	−0.053	0.628		
Others				
** **SRF at baseline	−0.074	0.493		
** **SRF at 1^st^ year	−0.044	0.691	−0.047	0.425
** **IRC at baseline	0.306	0.004^*∗*^		
** **IRC at 1^st^ year	−0.028	0.802	−0.08	0.284
** **RPED at baseline	0.067	0.538		
** **RPED at 1^st^ year	−0.02	0.855	0.166	0.011^*∗*^
Number of injections				
** **1^st^ year	−0.219	0.029^*∗*^		
** **>4 in 1^st^ year	−0.533	<0.001^*∗*^	−0.301	<0.001^*∗*^
** **Total	−0.293	0.003^*∗*^		

^*∗*^Statistically significant. SRF, subretinal fluid; IRC, intraretinal cyst; RPED, retinal pigment epithelial detachment.

**Table 2:** Univariate and multivariate analysis for central macular thickness at the third year in the complete 3-year follow-up group (*n* = 99).

*n* = 99	Univariate analysis		Multivariate analysis	
	*β*	*p* value	*β*	*p* value
Age	−0.032	0.765	−0.029	0.799
Sex	−0.044	0.685	0.004	0.969
Visual acuity				
** **Baseline	−0.062	0.567		
** **3^rd^ month	0.028	0.803		
** **1^st^ year	−0.092	0.401	−0.128	0.276
** **2^nd^ year	0	0.999		
Central macular thickness (CMT)				
** **Baseline	0.028	0.799		
** **3^rd^ month	0.306	0.008^*∗*^		
** **1^st^ year	0.258	0.031^*∗*^	0.341	0.034^*∗*^
** **2^nd^ year	0.434	<0.001^*∗*^		
** **3^rd^ year	0.589	<0.001^*∗*^		
Others				
** **SRF at baseline	0.152	0.187		
** **SRF at 1^st^ year	0.038	0.738	−0.130	−0.238
** **IRC at baseline	0.175	0.128		
** **IRC at 1^st^ year	0.374	0.001^*∗*^	0.161	0.275
** **RPED at baseline	−0.081	0.484		
** **RPED at 1^st^ year	0.210	0.066	0.021	0.857
Number of injections				
** **1^st^ year	−0.06	0.578	−0.034	0.765
** **Total	−0.059	0.586		

^*∗*^Statistically significant. SRF, subretinal fluid; IRC, intraretinal cyst; RPED, retinal pigment epithelial detachment.

## Data Availability

The data used to support the findings of this study are available from the corresponding author upon request.
